# A prognostic model for elderly patients with squamous non-small cell lung cancer: a population-based study

**DOI:** 10.1186/s12967-020-02606-3

**Published:** 2020-11-16

**Authors:** Siying Chen, Chunxia Gao, Qian Du, Lina Tang, Haisheng You, Yalin Dong

**Affiliations:** Department of Pharmacy, The First Affiliated Hospital of Xi’an Jiaotong University, No. 277 of Yanta West Road, Xi’an, 710061 Shaanxi China

**Keywords:** Nomogram, Squamous non-small cell lung cancer, Elderly patients, Survival prediction

## Abstract

**Background:**

Squamous cell carcinoma (SCC) is a main pathological type of non-small cell lung cancer. It is common among elderly patients with poor prognosis. We aimed to establish an accurate nomogram to predict survival for elderly patients (≥ 60 years old) with SCC based on the Surveillance, Epidemiology, and End Results (SEER) database.

**Methods:**

The gerontal patients diagnosed with SCC from 2010 to 2015 were collected from the Surveillance, Epidemiology, and End Results (SEER) database. The independent prognostic factors were identified using multivariate Cox proportional hazards regression analysis, which were utilized to conduct a nomogram for predicting survival. The novel nomogram was evaluated by Concordance index (C-index), calibration curves, net reclassification improvement (NRI), integrated discrimination improvement (IDI), and decision curve analysis (DCA).

**Results:**

32,474 elderly SCC patients were included in the analysis, who were randomly assigned to training cohort (n = 22,732) and validation cohort (n = 9742). The following factors were contained in the final prognostic model: age, sex, race, marital status, tumor site, AJCC stage, surgery, radiation and chemotherapy. Compared to AJCC stage, the novel nomogram exhibited better performance: C-index (training group: 0.789 vs. 0.730, validation group: 0.791 vs. 0.733), the areas under the receiver operating characteristic curve of the training set (1-year AUC: 0.846 vs. 0.791, 3-year AUC: 0.860 vs. 0.801, 5-year AUC: 0.859 vs. 0.794) and the validation set (1-year AUC: 0.846 vs. 0.793, 3-year AUC: 0.863 vs. 0.806, 5-year AUC: 0.866 vs. 0.801), and the 1-, 3- and 5-year calibration plots. Additionally, the NRI and IDI and 1-, 3- and 5-year DCA curves all confirmed that the nomogram was a great prognosis tool.

**Conclusions:**

We constructed a novel nomogram that could be practical and helpful for precise evaluation of elderly SCC patient prognosis, thus helping clinicians in determining the appropriate therapy strategies for individual SCC patients.

## Background

Lung cancer is the most frequent cause of cancer-related mortality in the world, with a 5-year survival of approximately 4–17% in populations [1, 2]. It is classified into two broad categories based on the different biological characteristics, treatment and prognosis: small cell lung cancer (SCLC, approximately 15% of all lung cancers) and non-small cell lung cancer (NSCLC, approximately 85% of all lung cancers) [3]. Among them, NSCLC is the most common type of lung cancer and has a poor prognosis, on account of initial diagnosis at an advanced stage (local or distant metastases) and a deficiency of effective treatment measures [4]. Adenocarcinoma (AC) and squamous cell carcinoma (SCC) are the two main pathological types of non-small cell lung cancer, and they account for approximately 40% and 30% of NSCLC cases, respectively [5].

SCC obviously differs in the biological characteristics from AC of the lung. It is observed that the classic SCC possesses the histological features including extensive areas of keratinization and intercellular bridges, whereas AC typically emerges gland formation and papillary structures, or solid growth with mucin production by microscope examination [6, 7]. Moreover, the differences between these two histologic subtypes are closely related to the site of origin. SCC is common centrally located, originating from early flat cells that align within the lung airways and appearing in the proximal bronchi. Inversely, AC is usually peripherally located and associated with surface alveolar epithelium or bronchial mucosa [6]. In recent years, the NSCLC patients have been greatly improved in treatment due to the alterations of driver genes, including epidermal growth factor receptor (EGFR) mutations, anaplastic lymphoma kinase (ALK) rearrangements, Kirsten rat sarcoma (KRAS) mutations, etc.[8]. However, the frequency of these genes mutations are low in SCC, and EGFR inhibitors, ALK inhibitors and other genes inhibitors are only effective in few of SCC patients. Consequently, the prognosis for patients with NSCLC is poorer for those with SCC than for those with AC.

In the evaluation of risk factors for SCC, a great number of studies have found smoking is the major risk factor for SCC patients. Nevertheless, smoking cessation can substantially decrease the risk for development of lung cancer and play a critical role in disease prevention and improved outcomes [6]. Moreover, in the male patients, SCC was the most common NSCLC histologic subtype [9]. In general, male can be used as a negative prognostic factor for NSCLC patients, several research data indicated that survival rates for female patients were significantly better than those for male patients [10]. Additionally, SCC patients tended to be slightly older than AC patients, and previous study suggested that 62% of SCC patients were 65 years or older at diagnosis, compared with 51% for elderly AC patients [11]. Furthermore, increased age is related to worse prognosis in NSCLC.

Clinically, in different histologic subtypes of NSCLC, SCC patients may have a worse prognosis and outcomes based on their molecular phenotype and driver oncogene. Currently, the American Joint Committee for Cancer (AJCC) staging system is a tool generally used by oncologists to predict tumor progression and develop therapeutic strategies [12]. However, considering the various risk factors affecting the development of NSCLC, it is obviously insufficient to predict the prognosis of patients only according to AJCC staging system. To our knowledge, no large-scale investigation has reported the relative significance of prognostic factors for SCC patients, especially for the elderly. Therefore, based on the abundance of patients information from the Surveillance, Epidemiology, and End Results (SEER) database, this study is aimed to establish a comprehensive prognostic evaluation model of elderly patients with SCC by building a nomogram to realize the risk factors and prognosis better. Moreover, we propose to compare the clinical usability and prognostic value of the nomogram with that of the AJCC staging system.

## Methods

### Patient selection and data processing

Patient data were obtained from the SEER database (covering 18 registries) using the SEER^*^ Stat version 8.3.5 (https://seer.cancer.gov/). We initially excluded other histologic subtypes of NSCLC, and selected 54,997 patients over 60 years of age who were diagnosed with squamous cell carcinoma between 2010 and 2015. The following variables were evaluated: age, sex, race, marital status (unmarried status included widowed, single, divorced and separated), tumor site, laterality, histology grade, AJCC stage, tumor size, metastatic sites, surgery, radiation, chemotherapy, insurance, follow-up time, cancer-specific death, and all-cause death. We excluded patients who did not have complete information on all the above variables (race unknown: n = 87, grade unknown: n = 20,104, stage unknown: n = 1428, surgery unknown: n = 174, radiation unknown: n = 331, and insurance unknown: n = 399). Ultimately, we identified 32,474 eligible SCC patients for this study. All data from the SEER database was free, and this study was approved by the Institutional Research Committee of the First Affiliated Hospital of Xi’an Jiaotong University.

### Nomogram establishment and validation

Firstly, we constructed a comprehensive prognostic nomogram. All SCC patients were randomly divided into training (n = 22,732) set and validation (n = 9742) set in a ratio of 7:3 [13, 14]. For the training cohort, we used univariate and multivariate Cox regression analysis to identify the prognostic factors, the hazard ratio (HR) and 95% confidence interval (CI) were calculated. The factors with statistical significance that affected lung cancer-specific survival (LCSS) and overall survival (OS) were included in the final prediction model. Then we established the nomogram for predicting 1-, 3- and 5-year survival rates in SCC patients using these identified prognostic factors.

Secondly, the nomogram was validated internally in the training group and externally in the validation group, respectively. We analyzed the concordance index (C-index), the receiver operating characteristic curve (ROC) and assessed the area under the curve (AUC) to evaluate the discriminative ability of the nomogram [15, 16]. Then the calibration curves were created to measure the correlation between the actual outcomes and the predictive performance [17]. Both discrimination and calibration were evaluated using 1000 bootstrap samples. We compared the accuracy of new prognostic model with that of traditional AJCC staging system using the net reclassification improvement (NRI) and the integrated discrimination improvement (IDI) [18]. Finally, the decision curve analysis (DCA) was preformed to assess the potential clinical usefulness and benefits of the predictive model [19, 20].

### Statistical analyses

Demographic and pathological features were compared using Chi-square test. The survival differences between different age groups were assessed using the Kaplan–Meier method. All statistical analyses were performed using SPSS 24.0 (SPSS Inc., Chicago, IL, USA) and R programming language (version 3.5.3; https://www.r-project.org/). And *P* value < 0.05 was considered statistically significant.

## Results

### Patient characteristics

A total of 32,474 SCC patients were finally included in this study, 22,732 patients constituted the training cohort and the other 9742 cases constituted the validation cohort. They were selected by a random split-sample method (split ratio: 7:3). Table 1 showed the detailed demographic data and clinicopathological characteristics of the two groups. The median age (25th–75th percentile) of this population was 73 (67–79) years. The median survival time (25th–75th percentile) was 10 (3–24) months. In the training cohort, elderly SCC cases tended to be male (61.1%), white (84.9%), upper lobe site (54.9%), grade III (53.1%) and stage I (31.5%) patients. Patient features were similar between the training set and the validation set.

**Table 1:** Patients' demographics and clinicopathological characteristics

Characteristics	Total cohort	Training cohort	Validation cohort	*P* value
	32,474 (100%)	22,732 (70.0%)	9742 (30.0%)	
Age				0.486
60–69	11,325 (34.9%)	7935 (34.9%)	3390 (34.8%)	
70–79	14,214 (43.8%)	9982 (43.9%)	4232 (43.4%)	
≥ 80	6935 (21.4%)	4815 (21.2%)	2120 (21.8%)	
Sex				0.456
Male	19,835 (61.1%)	13,848 (60.9%)	5987 (61.5%)	
Female	12,639 (38.9%)	8884 (39.1%)	3755 (38.5%)	
Race				0.059
White	27,573 (84.9%)	19,333 (85.1%)	8240 (84.6%)	
Black	3396 (10.5%)	2366 (10.4%)	1030 (10.6%)	
Asian or Pacific Islander	1320 (4.1%)	892 (3.9%)	428 (4.4%)	
American Indian/Alaska Native	185 (0.6%)	141 (0.6%)	44 (0.5%)	
Marital status				0.674
Married	16,673 (51.3%)	11,673 (51.4%)	5000 (51.3%)	
Unmarried	14,525 (44.7%)	10,152 (44.7%)	4373 (44.9%)	
Unknown	1276 (3.9%)	907 (4.0%)	369 (3.8%)	
Tumor site				0.665
Main bronchus	1353 (4.2%)	934 (4.1%)	419 (4.3%)	
Upper lobe, lung	17,829 (54.9%)	12,498 (55.0%)	5331 (54.7%)	
Middle lobe, lung	1254 (3.9%)	877 (3.9%)	377 (3.9%)	
Lower lobe, lung	10,212 (31.5%)	7154 (31.5%)	3058 (31.4%)	
Overlapping lesion of lung	358 (1.1%)	237 (1.0%)	121 (1.2%)	
Lung, NOS	1468 (4.5%)	1032 (4.5%)	436 (4.5%)	
Laterality				0.926
One side	32,203 (99.2%)	22,543 (99.2%)	9660 (99.2%)	
Bilateral	271 (0.8%)	189 (0.8%)	82 (0.8%)	
Grade				0.650
I	1023 (3.2%)	726 (3.2%)	297 (3.1%)	
II	13,902 (42.8%)	9704 (42.7%)	4198 (43.1%)	
III	17,252 (53.1%)	12,101 (53.2%)	5151 (52.9%)	
IV	297 (0.9%)	201 (0.9%)	96 (1.0%)	
AJCC stage				0.679
I	10,216 (31.5%)	7193 (31.6%)	3023 (31.0%)	
II	4970 (15.3%)	3480 (15.3%)	1490 (15.3%)	
III	8313 (25.6%)	5812 (25.6%)	2501 (25.7%)	
IV	8975 (27.6%)	6247 (27.5%)	2728 (28.0%)	
Tumor size				0.302
≤ 30	10,783 (33.2%)	7569 (33.3%)	3214 (33.0%)	
31–50	8669 (26.7%)	6117 (26.9%)	2552 (26.2%)	
51–70	5770 (17.8%)	4001 (17.6%)	1769 (18.2%)	
> 70	4650 (14.3%)	3258 (14.3%)	1392 (14.3%)	
Unknown	2602 (8.0%)	1787 (7.9%)	815 (8.4%)	
Bone metastasis				0.148
Yes	2475 (7.6%)	1715 (7.5%)	760 (7.8%)	
No	29,558 (91.0%)	20,691 (91.0%)	8867 (91.0%)	
Unknown	441 (1.4%)	326 (1.4%)	115 (1.2%)	
Brain metastasis				0.433
Yes	1255 (3.9%)	863 (3.8%)	392 (4.0%)	
No	30,764 (94.7%)	21,542 (94.8%)	9222 (94.7%)	
Unknown	455 (1.4%)	327 (1.4%)	128 (1.3%)	
Liver metastasis				0.199
Yes	1325 (4.1%)	909 (4.0%)	416 (4.3%)	
No	30,707 (94.5%)	21,500 (94.6%)	9207 (94.5%)	
Unknown	442 (1.4%)	323 (1.4%)	119 (1.2%)	
Lung metastasis				0.573
Yes	2993 (9.2%)	2101 (9.2%)	892 (9.2%)	
No	28,973 (89.2%)	20,286 (89.2%)	8687 (89.2%)	
Unknown	508 (1.6%)	345 (1.5%)	163 (1.7%)	
Surgery				0.492
Yes	12,009 (37.0%)	8379 (36.9%)	3630 (37.3%)	
No	20,465 (63.0%)	14,353 (63.1%)	6112 (62.7%)	
Radiation				0.443
Yes	13,393 (41.2%)	9344 (41.1%)	4049 (41.6%)	
No	19,081 (58.8%)	13,388 (58.9%)	5693 (58.4%)	
Chemotherapy				0.865
Yes	11,796 (36.3%)	8264 (36.4%)	3532 (36.3%)	
No	20,678 (63.7%)	14,468 (63.6%)	6210 (63.7%)	
Insurance				0.377
Yes	28,421 (87.5%)	19,919 (87.6%)	8502 (87.3%)	
No	4053 (12.9%)	2813 (12.4%)	1240 (12.7%)	
Median follow-up time (Months, 25th–75th percentile)	10 (3–24)	10 (3–25)	10 (3–24)	0.879

*AJCC* The American Joint Committee for Cancer

### Prognostic predictors for elderly SCC

**Fig. 1:**
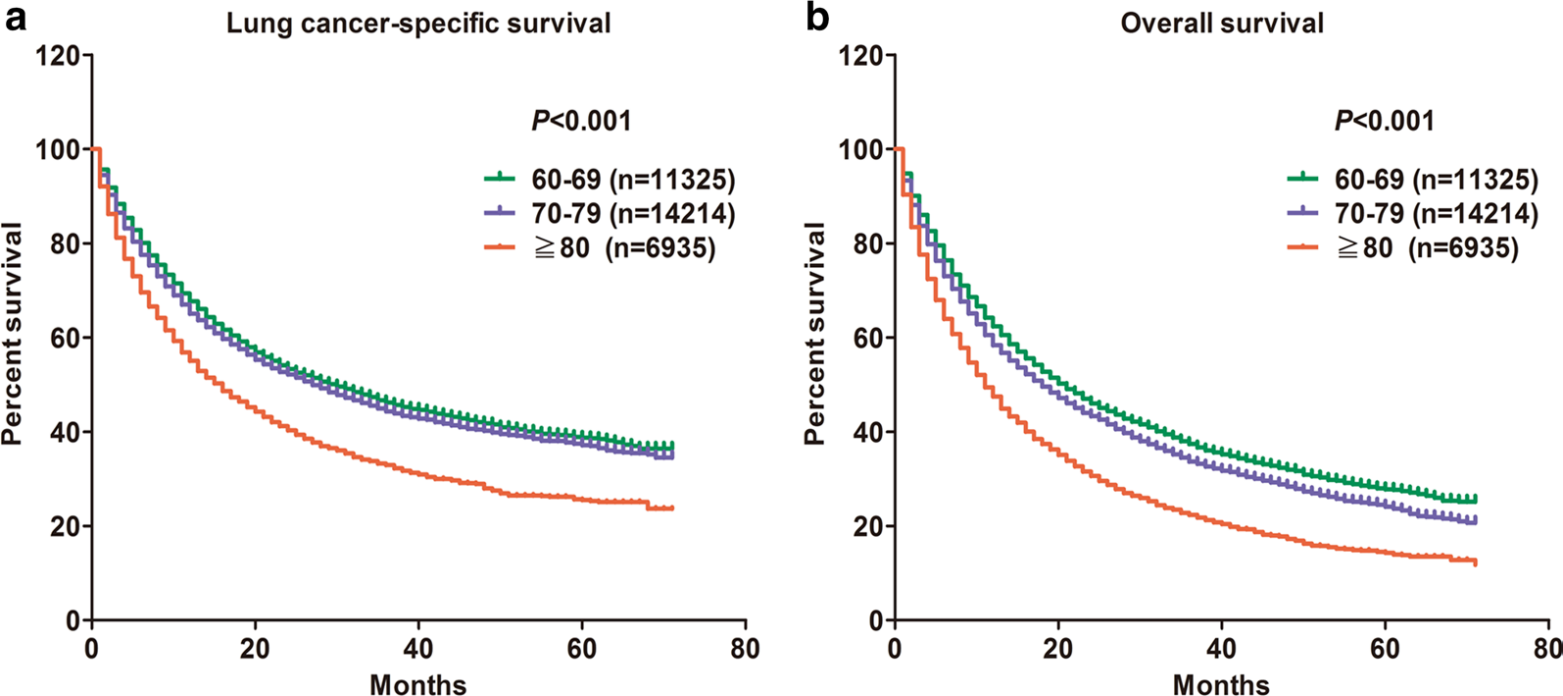
The effect of age at diagnosis on the lung cancer-specific survival and overall survival of elderly patients with SCC. Kaplan–Meier curves for **a** lung cancer-specific survival (*P* < 0.001), and **b** overall survival (*P* < 0.001)

Firstly, we performed OS and LCSS analysis among different age groups and found that the poor survival outcomes were mainly concentrated in SCC patients with the age of diagnosis ≥ 80 years (Fig. 1). Afterwards, for the training cohort, we identified nine independent prognostic factors based on the univariate and multivariate Cox proportional hazards regression analysis. It was observed that age at diagnosis, sex, race, marital status, tumor site, AJCC stage, surgery, radiation and chemotherapy were all significantly associated with LCSS in elderly patients with SCC (Table 2). Among these factors, the risk of age (≥ 80) at diagnosis (HR = 1.185, *P* < 0.001), AJCC stage II (HR = 2.544, *P* < 0.001), AJCC stage III (HR = 3.689, *P* < 0.001), AJCC stage IV (HR = 6.245, *P* < 0.001), no surgery (HR = 3.643, *P* < 0.001), no radiation (HR = 1.483, *P* < 0.001), and no chemotherapy (HR = 1.812, *P* < 0.001) were higher than other factors. The related data for OS of SCC patients was in Additional file 1: Table S1.

**Table 2:** Univariate and multivariate Cox regression analysis based on all variables for cancer-specific survival (Training Cohort)

Characteristics	Univariate analysis		Multivariate analysis	
	HR (95% CI)	*P* value	HR (95% CI)	*P* value
Age				
60–69	Reference		Reference	
70–79	1.074 (1.028–1.122)	0.001	1.075 (1.029–1.124)	0.001
≥ 80	1.511 (1.437–1.588)	< 0.001	1.185 (1.124–1.249)	< 0.001
Sex				
Male	Reference		Reference	
Female	0.871 (0.838–0.905)	< 0.001	0.862 (0.828–0.898)	< 0.001
Race				
White	Reference		Reference	
Black	1.115 (1.049–1.184)	< 0.001	0.884 (0.831–0.940)	< 0.001
Asian or Pacific Islander	1.152 (1.048–1.265)	0.003	0.880 (0.800–0.968)	0.008
American Indian/Alaska Native	1.110 (0.876–1.407)	0.388	1.039 (0.819–1.317)	0.754
Marital				
Married	Reference		Reference	
Unmarried	1.146 (1.103–1.190)	< 0.001	1.042 (1.000–1.085)	0.049
Unknown	0.996 (0.901–1.102)	0.944	0.945 (0.853–1.046)	0.273
Site				
Main bronchus	Reference		Reference	
Upper lobe, lung	0.476 (0.438–0.517)	< 0.001	0.717 (0.660–0.779)	< 0.001
Middle lobe, lung	0.480 (0.424–0.544)	< 0.001	0.768 (0.677–0.871)	< 0.001
Lower lobe, lung	0.484 (0.445–0.528)	< 0.001	0.737 (0.676–0.804)	< 0.001
Overlapping lesion of lung	0.552 (0.453–0.672)	< 0.001	0.866 (0.710–1.055)	0.152
Lung, NOS	1.019 (0.914–1.135)	0.735	0.797 (0.712–0.892)	< 0.001
Laterality				
One side	Reference		Reference	
Bilateral	2.393 (2.006–2.855)	< 0.001	0.890 (0.738–1.074)	0.224
Grade				
I	Reference		Reference	
II	0.895 (0.801–0.999)	0.048	0.962 (0.861–1.074)	0.492
III	1.104 (0.990–1.232)	0.075	1.013 (0.908–1.130)	0.814
IV	1.288 (1.036–1.600)	0.023	1.112 (0.894–1.382)	0.340
AJCC stage				
I	Reference		Reference	
II	2.257 (2.099–2.426)	< 0.001	2.544 (2.363–2.738)	< 0.001
III	4.080 (3.839–4.336)	< 0.001	3.689 (3.447–3.948)	< 0.001
IV	8.800 (8.294–9.337)	< 0.001	6.245 (5.830–6.691)	< 0.001
Surgery				
Yes	Reference		Reference	
No	5.003 (4.759–5.259)	< 0.001	3.643 (3.421–3.879)	< 0.001
Radiation				
Yes	Reference		Reference	
No	0.798 (0.768–0.828)	< 0.001	1.483 (1.421–1.548)	< 0.001
Chemotherapy				
Yes	Reference		Reference	
No	0.871 (0.839–0.905)	< 0.001	1.812 (1.736–1.892)	< 0.001
Insurance				
Yes	Reference		Reference	
No	1.228 (1.162–1.297)	< 0.001	1.033 (0.977–1.094)	0.255

*AJCC* The American Joint Committee for Cancer

### Development of elderly SCC nomogram

**Fig. 2:**
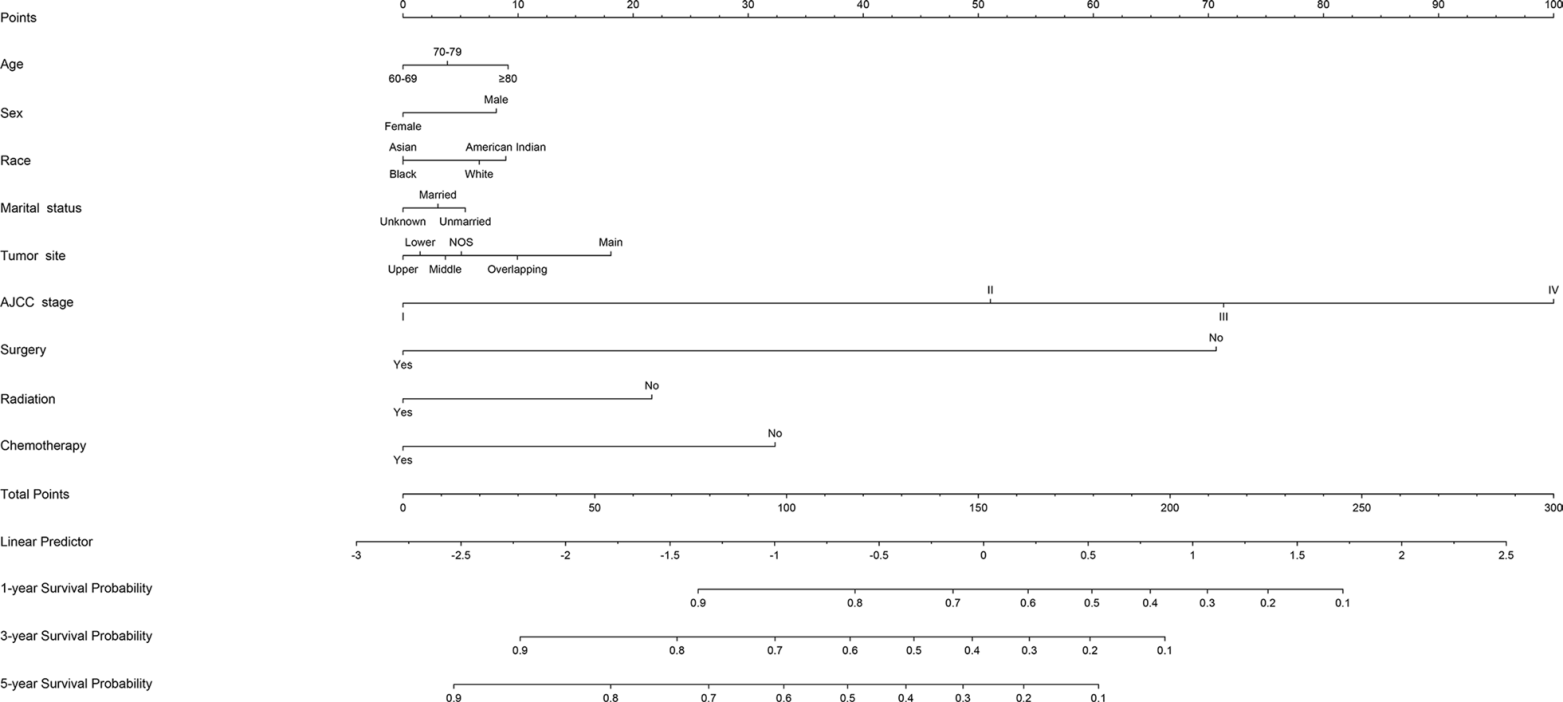
Nomogram predicted 1-, 3- and 5-year lung cancer-specific survival for elderly SCC patients with nine available factors, including age, sex, race, marital status, tumor site, the American Joint Committee for Cancer (AJCC) stage, surgery, radiation and chemotherapy

A nomogram model based on nine selected prognostic predictors from the training cohort was developed for the 1-, 3-, and 5-year survival prediction of LCSS in elderly SCC patients (Fig. 2). The nomogram illustrated the points of each predictor ranging from 0 to 100. The results demonstrated that AJCC stage score as sharing the most contribution to prognosis, followed by surgery, chemotherapy, and radiation. The total scores were calculated and located on the total point scale by adding the scores for each predictor. The prediction probabilities corresponding to this total score was able to estimate the 1-, 3- and 5-year LCSS for each individual patient. The nomogram model for OS were shown in Additional file 1: Figure S1.

### Calibration and validation of the nomogram

**Fig. 3:**
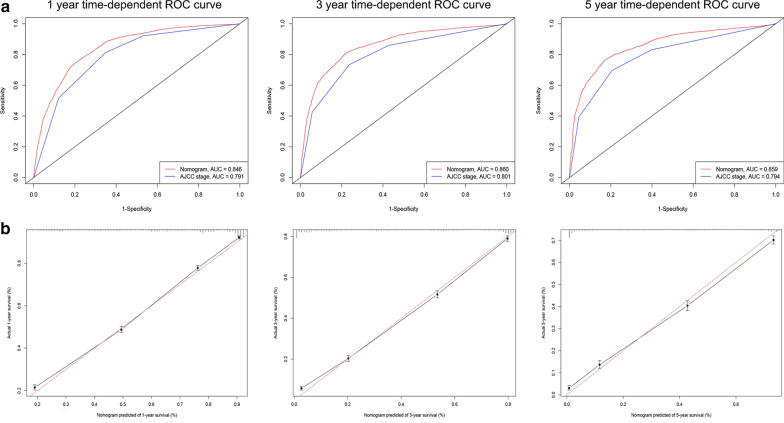
ROC curves and calibration plots for predicting 1-, 3- and 5-year lung cancer-specific survival for elderly SCC patients in the training cohorts. **a** ROC curves of the Nomogram and AJCC stage in prediction of prognosis at 1-, 3- and 5-year point in the training set. **b** The calibration plots for predicting patient survival at 1-, 3- and 5-year point in the training set. *ROC* receiver operating characteristic curve, *AUC* areas under the ROC curve

**Fig. 4:**
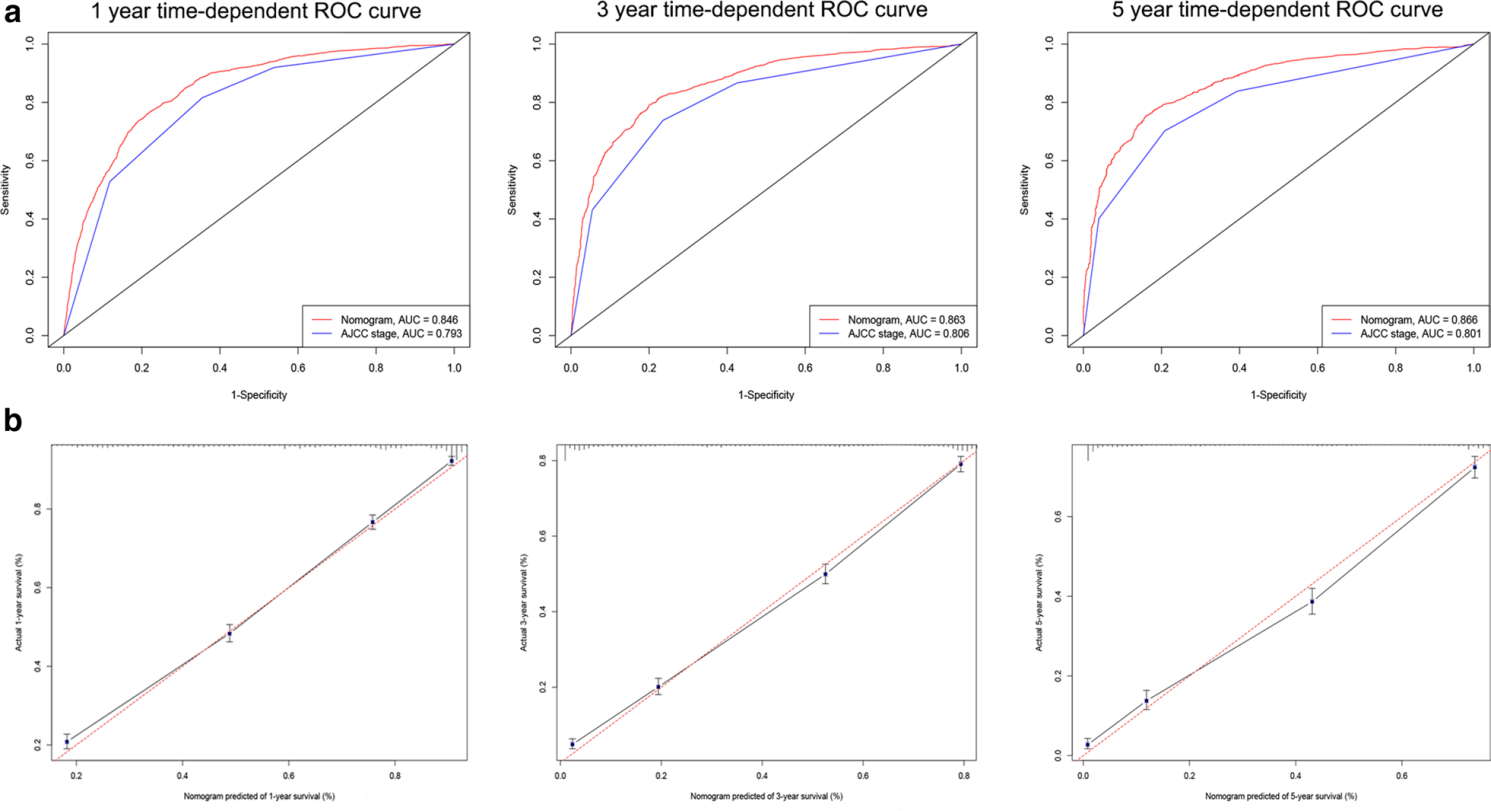
ROC curves and calibration plots for predicting 1-, 3- and 5-year lung cancer-specific survival for elderly SCC patients in the validation cohorts. **a** ROC curves of the Nomogram and AJCC stage in prediction of prognosis at 1-, 3- and 5-year point in the validation set. **b** The calibration plots for predicting patient survival at 1-, 3- and 5-year point in the validation set. *ROC* receiver operating characteristic curve, *AUC* areas under the ROC curve

The nomogram exhibited quite accuracy in estimating the survival of gerontal patients with SCC, with higher C-indices (training cohort = 0.789, validation cohort = 0.791) than those based on the AJCC stage (training cohort = 0.730, validation cohort = 0.733). Moreover, for predictions of 1-, 3- and 5-year LCSS, the AUC of nomogram is higher than that of AJCC stage both in the training group (1-year: 0.846 vs. 0.791, 3-year: 0.860 vs. 0.801, 5-year: 0.859 vs. 0.794, Fig. 3a) and validation group (1-year: 0.846 vs. 0.793, 3-year: 0.863 vs. 0.806, 5-year: 0.866 vs. 0.801, Fig. 4a). These results intensively indicated that the nomogram model had good predicting ability and discrimination. In addition, calibration plots presented a good agreement between the nomogram predicted outcomes and actual observations for predicting 1-, 3- and 5-year LCSS in the training (Fig. 3b) and validation (Fig. 4b) cohorts. The related results for OS were listed in Additional file 1: Figures S2, S3.

The NRI values in the validation cohort were 0.389 (95% CI 0.331–0.464), 0.454 (95% CI 0.295–0.580), and 0.454 (95% CI 0.386–0.536) for 1, 3, and 5 years of follow-up, respectively. Similarly, in the validation set, the IDI for 1-, 3- and 5-year of follow-up were 0.060 (*P* < 0.001), 0.056 (*P* < 0.001) and 0.048 (*P* < 0.001), respectively. These findings validated that the nomogram model exhibited superior predictive performance compared to the AJCC staging model.

### Clinical value of the nomogram

**Fig. 5:**
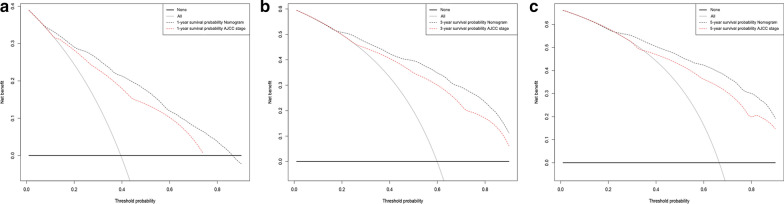
Decision curve analysis for the Nomogram and AJCC stage in prediction of prognosis of elderly SCC patients at 1-year (**a**), 3-year (**b**) and 5-year (**c**) point in the validation cohorts

DCA is a novel method for evaluating prognostic decision-making, which has some advantages over AUC [21]. The DCA curves of new nomogram and AJCC staging model for predicting the 1-, 3-, and 5-year LCSS rates in the validation groups are presented in Fig. 5. The new nomogram showed higher net benefits across a range of death risk compared with the AJCC stage, which indicated that it had better clinical utility. The related results for OS were listed in Additional file 1: Figure S4.

## Discussion

With the development of molecularly targeted therapy, although it has revolutionized the treatment of patients with EGFR mutation-positive NSCLC, the incidence of this activating mutation of EGFR is lower in SCC patients than in AC patients, which limits the effect of clinical treatment for SCC cases. Moreover, SCC patients tend to be older than those with AC [22]. Ultimately, the prognosis for gerontal patients with advanced NSCLC is poorer for those with SCC than for those with AC [6, 23]. Therefore, it is necessary to construct a model to assess the prognosis for elderly SCC patients, to assist clinicians in making therapeutic strategies for these patients. In general, clinical AJCC staging system plays a crucial role in predicting survivor as well as influencing management option in NSCLC patients. However, it neglects various significant risk factors such as age, race, and potential markers [12]. In the current study, we established a more comprehensive model based on a combination of significant risk factors to predict the survival probability for individual elderly SCC patients. This nomogram included nine variables: age, sex, race, marital status, tumor site, AJCC stage, surgery, radiation and chemotherapy, which was capable of making more accurate evaluations and predictions in elderly SCC patients compared to the traditional AJCC staging system both in the training and validation groups.

The previous study reported that SCC was the predominant NSCLC histologic subtype in men [9, 22]. We also found that elderly SCC cases tended to be male sex in our investigation. Moreover, it was observed that the proportion of patients with white, upper lobe site, grade III and stage I were quite large in the corresponding variables. These were also unique characteristics of elderly SCC patients. In addition, we identified nine risk factors that have an impact on the elderly SCC-specific survival. The independent prognostic factors in our study were similar to those identified in several previous researches. Gu et al. has discovered that increasing age and histopathologic N2 disease can be correlated with poor LCSS for lung squamous cell carcinoma patients [22]. A study by Ryota et al. has identified that increased tumor size, lymph node metastasis, lymphatic invasion, poor tissue differentiation and INFc(−) can be significantly associated with increased mortality of lung squamous cell carcinoma [24]. Moreover, ten variables including age, sex, histology, grade, lymph node examined, positive lymph node, visceral pleural invasion, tumor size, therapy type, and surgery type were observably correlated with postoperative survival of stage IIIA-N2 NSCLC [25]. The findings of our analysis showed that age, AJCC stage, surgery, radiation and chemotherapy had a greater impact on the prognosis of elderly SCC patients.

Although the effect of AJCC staging system on prognosis is quite important, it might not comprehensively predict the prognosis of patients. Therefore, considering the influence of the above-described risk factors, we firstly developed a relatively comprehensive nomogram for predicting elderly SCC-specific survival. In our new established model, it is clear that AJCC stage and surgery have a significant impact on the total score used for predicting the outcomes of elderly SCC patients. These findings were similar with previous reports, it was observed that metastatic location and pathological grading were crucial independent predictors for OS in NSCLC [26]. Some studies discovered that chemotherapy, metastasis and surgery made the larger contribution to the prognosis for distantly metastatic non-small-cell lung cancer [27, 28]. Additionally, we also found that the tumor site of main bronchus had higher risk scores implying that these elderly SCC patients had a poor prognosis, which is consistent with findings in previous reports [29, 30]. Moreover, the grade was supposed to possess a certain correlation with the patient's prognosis. But in the multivariate Cox regression analysis, the grade was not a prognostic factor. This implied that taking active treatments, such as surgery, radiotherapy and chemotherapy, may weaken the impact of grade on the prognosis of patients. Ultimately, our results indicated that the C-index, ROC curve and calibration curve of predictive model were great in the validation cohort, implying that this nomogram had good predictive accuracy and reliability.

Next, we evaluated the performance and clinical practice of newly established model by NRI and IDI analysis [31, 32]. Compared with the AJCC staging system, our nomogram had better accuracy and discriminability for predicting 1-, 3- and 5-year LCSS of elderly SCC patients. As such, to assess the net benefit of this prediction model across a range of threshold risks to facilitate clinical decision, we proved that the newly established nomogram predicted survival with much more practical and high-efficiency than the AJCC staging system by using DCA. Similarly, several studies have applied DCA to evaluate if nomogram assisted decisions improve patient outcomes [33–35]. To the best of our knowledge, this is the large-population study to construct a comprehensive and intensive nomogram for gerontal patients with SCC. Comparing with traditional AJCC staging system, our newly established model demonstrates better ability and value for elderly SCC patients. It is believed that this well-designed nomogram can be used to predict the prognosis of each individual patient and thereby brings benefits to both clinicians and patients.

Similar to other reports using the SEER database, we had some limitations. First, the retrospective nature of the data collection from the SEER database may lead to some inherent and selection biases. Second, during the process of analysis, several missing data were excluded on the collected variables, which inevitably have selection bias. Third, the important prognostic factors of NSCLC, such as the family history of lung cancer, smoking status, the surgical margin status, vascular invasion, genotype characteristics and the detailed information of drug therapy were not provided by the SEER database. Additionally, co-morbid conditions could affect the disease and response to treatment of SCC patients, especially in the older population group. In the future, more potential influencing factors should be included in the model to achieve more comprehensive predictive ability for the prognosis of elderly SCC patients. Finally, our nomogram is only concluded based on the data from the patients in the USA and thus, may not be representative of the SCC patients worldwide. In the following research, it is important to verify the accuracy and practicality of this model by external validation using other populations with SCC. Meanwhile, we will continue to optimize and improve this model during the clinical application, hoping to finally get a better prognosis model for patients with lung squamous cell carcinoma.

## Conclusion

In conclusion, we developed a novel nomogram that could more accurately predict the prognosis of elderly patients with SCC. The proposed nomogram integrated the influential clinical predictors and revealed a significant improvement in the prediction of elderly SCC patients’ survival compared with traditional AJCC staging system. We have confirmed the excellent discrimination and clinical usability of this nomogram by comparing it to the AJCC staging system. These results provide a more precise reference and a practical tool for the accurate prognosis prediction of SCC in clinical.

## Supplementary information

**Additional file 1: Table S1.** Univariate and multivariate Cox regression analysis based on all variables for overall survival (Training Cohort). **Figure S1.** Nomogram predicted 1-, 3- and 5-year overall survival for elderly SCC patients with ten available factors, including age, sex, race, marital status, tumor site, the American Joint Committee for Cancer (AJCC) stage, surgery, radiation, chemotherapy and Insurance. **Figure S2.** ROC curves and calibration plots for predicting 1-, 3- and 5-year overall survival for elderly SCC patients in the training cohorts. (A) ROC curves of the Nomogram and AJCC stage in prediction of prognosis at 1-, 3- and 5-year point in the training set. (B) The calibration plots for predicting patient survival at 1-, 3- and 5-year point in the training set. ROC: receiver operating characteristic curve; AUC: areas under the ROC curve. **Figure S3.** ROC curves and calibration plots for predicting 1-, 3- and 5-year overall survival for elderly SCC patients in the validation cohorts. (A) ROC curves of the Nomogram and AJCC stage in prediction of prognosis at 1-, 3- and 5-year point in the validation set. (B) The calibration plots for predicting patient survival at 1-, 3- and 5-year point in the validation set. ROC: receiver operating characteristic curve; AUC: areas under the ROC curve. **Figure S4.** Decision curve analysis for the Nomogram and AJCC stage in prediction of overall survival of elderly SCC patients at 1-year (A), 3-year (B) and 5-year (C) point in the validation cohorts.

## Data Availability

The datasets analyzed in this study are available in the SEER repository and can be obtained from: https://seer.cancer.gov/data/.

## Abbreviations

NSCLC: Non-small cell lung cancer
SCLC: Small cell lung cancer
SCC: Squamous cell carcinoma
EGFR: Epidermal growth factor receptor
ALK: Anaplastic lymphoma kinase
KRAS: Kirsten rat sarcoma
AJCC: American Joint Committee for Cancer
SEER: Surveillance, Epidemiology, and End Results
LCSS: Lung cancer-specific survival
OS: Overall survival
C-index: Concordance index
ROC: Receiver operating characteristic curve
AUC: Area under the curve
NRI: Net reclassification improvement
IDI: Integrated discrimination improvement
DCA: Decision curve analysis
