# Supplementary material for: A prognostic model for elderly patients with squamous non-small cell lung cancer: a population-based study
Source: J Transl Med. 2020 Nov 16;18:436. doi: 10.1186/s12967-020-02606-3 (PMC7670679; doi:10.1186/s12967-020-02606-3)
Supplement: Supplementary file 1 — Additional file 1: Table S1. Univariate and multivariate Cox regression analysis based on all variables for overall survival (Training Cohort). Figure S1. Nomogram predicted 1-, 3- and 5-year overall survival for elderly SCC patients with ten available factors, including age, sex, race, marital status, tumor site, the American Joint Committee for Cancer (AJCC) stage, surgery, radiation, chemotherapy and Insurance. Figure S2. ROC curves and calibration plots for predicting 1-, 3- and 5-year overall survival for elderly SCC patients in the training cohorts. (A) ROC curves of the Nomogram and AJCC stage in prediction of prognosis at 1-, 3- and 5-year point in the training set. (B) The calibration plots for predicting patient survival at 1-, 3- and 5-year point in the training set. ROC: receiver operating characteristic curve; AUC: areas under the ROC curve. Figure S3. ROC curves and calibration plots for predicting 1-, 3- and 5-year overall survival for elderly SCC patients in the validation cohorts. (A) ROC curves of the Nomogram and AJCC stage in prediction of prognosis at 1-, 3- and 5-year point in the validation set. (B) The calibration plots for predicting patient survival at 1-, 3- and 5-year point in the validation set. ROC: receiver operating characteristic curve; AUC: areas under the ROC curve. Figure S4. Decision curve analysis for the Nomogram and AJCC stage in prediction of overall survival of elderly SCC patients at 1-year (A), 3-year (B) and 5-year (C) point in the validation cohorts. [file 12967_2020_2606_MOESM1_ESM.pdf]

Table S1 Univariate and multivariate Cox regression analysis based on all variables for overall survival (Training Cohort)

Characteristics	Univariate analysis		Multivariate analysis	
	HR (95% CI)	*P* value	HR (95% CI)	*P* value
**Age**				
60 - 69	Reference		Reference	
70 - 79	1.115（1.073-1.159）	＜0.001	1.106（1.063-1.150）	＜0.001
≥ 80	1.562（1.495-1.633）	＜0.001	1.224（1.168-1.283）	＜0.001
**Sex**				
Male	Reference		Reference	
Female	0.857（0.828-0.887）	＜0.001	0.833（0.804-0.864）	＜0.001
**Race**				
White	Reference		Reference	
Black	1.127（1.068-1.188）	＜0.001	0.914（0.865-0.964）	0.001
Asian or Pacific Islander	1.097（1.008-1.195）	0.033	0.853（0.782-0.930）	＜0.001
American Indian/Alaska Native	1.147（0.934-1.410）	0.191	1.080（0.878-1.327）	0.467
**Marital**				
Married	Reference		Reference	
Unmarried	1.158（1.119-1.198）	＜0.001	1.060（1.022-1.099）	0.002
Unknown	1.016（0.930-1.110）	0.722	0.972（0.889-1.062）	0.526
**Site**				
Main bronchus	Reference		Reference	
Upper lobe, lung	0.523（0.485-0.564）	＜0.001	0.744（0.688-0.803）	＜0.001
Middle lobe, lung	0.526（0.470-0.588）	＜0.001	0.786（0.702-0.880）	＜0.001
Lower lobe, lung	0.534（0.494-0.578）	＜0.001	0.764（0.706-0.828）	＜0.001
Overlapping lesion of lung	0.548（0.457-0.657）	＜0.001	0.828（0.690-0.994）	0.043
Lung, NOS	1.079（0.977-1.192）	0.134	0.837（0.755-0.928）	0.001
**Laterality**				
One side	Reference		Reference	
Bilateral	2.422（2.070-2.833）	＜0.001	0.975（0.825-1.152）	0.765
**Grade**				
I	Reference		Reference	
II	0.889（0.807-0.979）	0.017	0.945（0.858-1.040）	0.246
III	1.055（0.960-1.161）	0.267	0.979（0.890-1.077）	0.661
IV	1.210（0.997-1.470）	0.054	1.070（0.881-1.300）	0.495
**AJCC stage**				
I	Reference		Reference	
II	1.710（1.612-1.813）	＜0.001	1.970（1.855-2.092）	＜0.001
III	2.855（2.719-2.999）	＜0.001	2.746（2.597-2.903）	＜0.001
IV	5.982（5.703-6.274）	＜0.001	4.541（4.290-4.807）	＜0.001
**Surgery**				
Yes	Reference		Reference	
No	3.958（3.800-4.124）	＜0.001	3.275（3.106-3.454）	＜0.001
**Radiation**				

Yes	Reference		Reference	
No	0.829（0.801-0.857）	＜0.001	1.482（1.426-1.540）	＜0.001
**Chemotherapy**				
Yes	Reference		Reference	
No	0.974（0.942-1.008）	0.138	1.863（1.792-1.936）	＜0.001
**Insurance**				
Yes	Reference		Reference	
No	1.236（1.177-1.297）	＜0.001	1.063（1.011-1.118）	0.016

AJCC: The American Joint Committee for Cancer

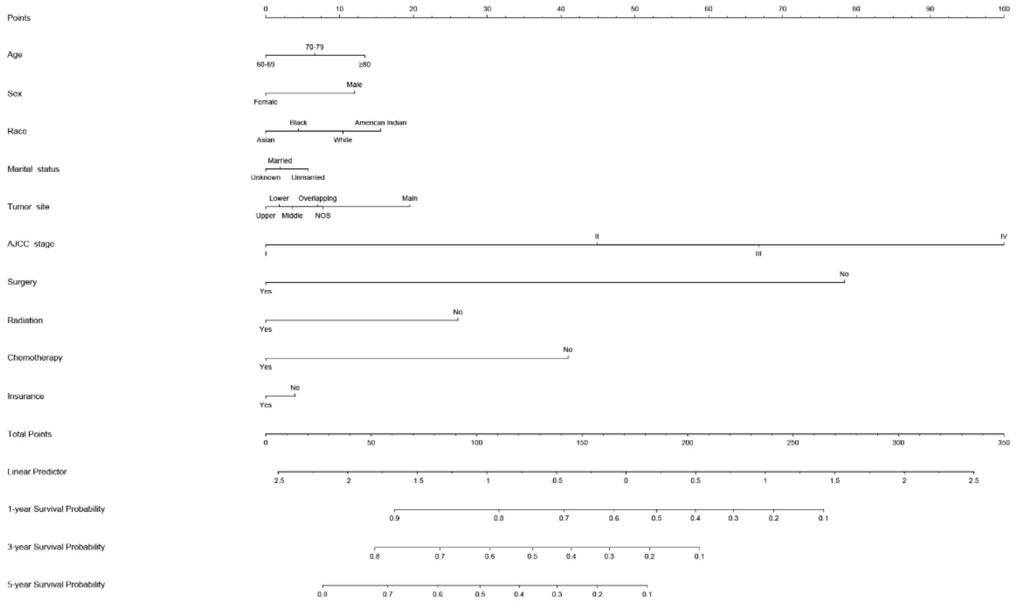

Figure S1

Figure S1 Nomogram predicted 1-, 3- and 5-year overall survival for elderly SCC patients with ten available factors, including age, sex, race, marital status, tumor site, the American Joint Committee for Cancer (AJCC) stage, surgery, radiation, chemotherapy and Insurance.

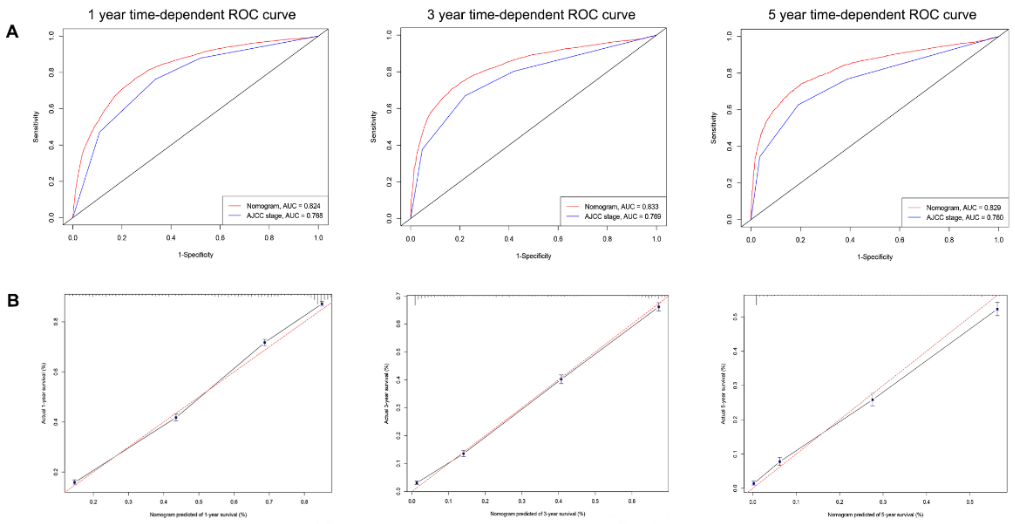

Figure S2

Figure S2 ROC curves and calibration plots for predicting 1-, 3- and 5-year overall survival for elderly SCC patients in the training cohorts. (A) ROC curves of the Nomogram and AJCC stage in prediction of prognosis at 1-, 3- and 5-year point in the training set. (B) The calibration plots for predicting patient survival at 1-, 3- and 5-year point in the training set. ROC: receiver operating characteristic curve; AUC: areas under the ROC curve.

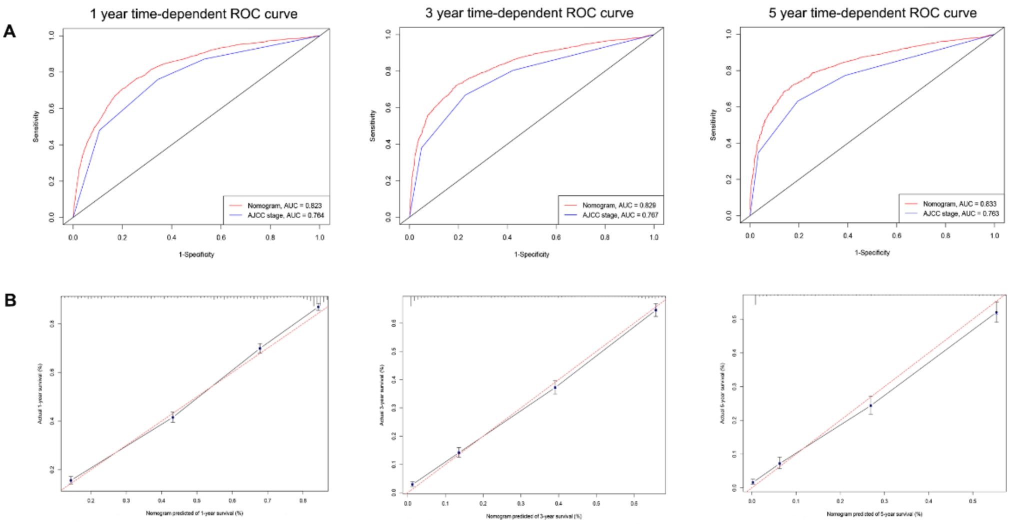

Figure S3

Figure S3 ROC curves and calibration plots for predicting 1-, 3- and 5-year overall survival for elderly SCC patients in the validation cohorts. (A) ROC curves of the Nomogram and AJCC stage in prediction of prognosis at 1-, 3- and 5-year point in the validation set. (B) The calibration plots for predicting patient survival at 1-, 3- and 5-year point in the validation set. ROC: receiver operating characteristic curve; AUC: areas under the ROC curve.

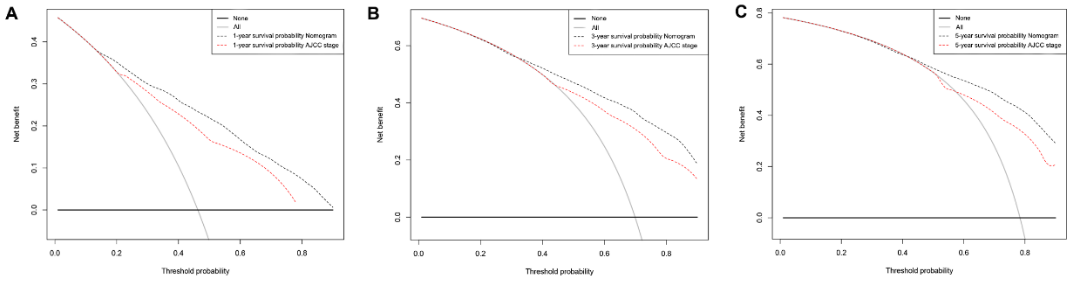

Figure S4

Figure S4 Decision curve analysis for the Nomogram and AJCC stage in prediction of overall survival of elderly SCC patients at 1-year (A), 3-year (B) and 5-year (C) point in the validation cohorts.
